# Editorial: Local ablative therapies for the management of lung cancer

**DOI:** 10.3389/fonc.2023.1160932

**Published:** 2023-02-16

**Authors:** Zhigang Wei, Roberto Iezzi, Xin Ye

**Affiliations:** ^1^ Department of Oncology, The First Affiliated Hospital of Shandong First Medical University & Shandong Provincial Qianfoshan Hospital, Shandong Lung Cancer Institute, Shandong Key Laboratory of Rheumatic Disease and Translational Medicine, Jinan, China; ^2^ Department of Diagnostic Imaging, Oncologic Radiotherapy and Hematology-A. Gemelli University Hospital Foundation IRCCS, Rome, Italy; ^3^ Istituto di Radiodiagnostica, Università Cattolica del Sacro Cuore, Rome, Italy

**Keywords:** lung cancer, metastases, ablation, MRI, combination

Lung cancer, the leading cause of cancer related death in China and worldwide (1, 2), were comprised of non-small cell lung cancer (NSCLC) and small cell lung cancer (SCLC), and the former account for nearly 85% (3). Furthermore, lungs have become the second most common metastatic site after liver, having a great impact on patient management and outcome (4). Therefore, the diagnosis and treatment of primary and secondary lung tumors deserves high emphasis.

For early-stage NSCLC and for some lung metastases, radical surgery usually represents the main treatment method; in unsurgical patients, percutaneous image-guided thermal ablation, such as radiofrequency ablation (RFA), microwave ablation (MWA) and cryoablation, has been increasingly applied as a safe, cost- effective, and precise minimally invasive alternative treatment methods (5).

Based on recent technical developments and new combinations of systemic and local therapy, we recognized the topic of “Local Ablative Therapies for The Management of Lung Cancer“ in Frontiers of Oncology.

## Primary lung cancer

Recently with application of low dose CT (LDCT), more patients with ground-glass nodules (GGNs) were identified and part GGNs progress to GGN-like lung adenocarcinoma. Although surgery was the main treatment regimen for those patients, MWA have being proved to be another radical treatment option (6, 7). However, up to date, no study explored the cost and effectiveness of microwave ablation versus video-assisted thoracoscopic surgical (VATS) resection for GGN-like lung adenocarcinoma. Han et al. verified that MWA had similar 3-year overall survival (OS) rate and a dramatically lower cost and shorter hospital stay compared with VATS. So based on efficacy and cost, MWA could provide an alternative treatment option for patients with GGN-like lung adenocarcinoma.

Although MWA was mainly applied in early-stage NSCLC, for locally advanced or metastatic NSCLC patients, MWA in combination with other treatments could also improve survival in the study. Based on the SEER database, Yang et al. verified that MWA improved both OS and cancer specific survival (CSS) for patients with stage II to III NSCLC, which indicating MWA maybe an alternative treatment for those unfit for radical surgery.

For advanced and standard treatments-refractory NSCLC patients, local treatment of drug-eluting beads bronchial arterial chemoembolization (DEB-BACE) could be an option (8, 9), also for treating complications. When DEB-BACE was combined with MWA, superior ORR and longer PFS were achieved as showed by Xu et al. Other combination treatment regimens such as DEB-BACE plus targeted therapy of anlotinb or iodine-125 brachytherapy also showed response and survival advantage (10, 11). The combination of systematic treatments of chemotherapy with local treatment such as radiotherapy and interventional embolization could achieve superior survival advantage. Zhou et al. reported a partial response after combined treatment in a squamous cell unresectable lung cancer was also achieved.

The treatments for advanced NSCLC range from routine chemotherapy to novel targeted therapy and immunotherapy (12). Immunotherapy targeting immune checkpoint (IC) especially anti-programmed death-1 (PD-1) and programmed death-ligand 1(PD-L1) antibodies have changed the treatment regimens of metastatic NSCLC. However, the relatively low response rate of immunotherapy monotherapy limits its application. Our previous study verified that PD-1 antibody combined with MWA could improve ORR (13). We further explored the MWA plus camrelizumab (a PD-1 inhibitor) monotherapy or camrelizumab combination therapy in NSCLC. The ORR was 29.9% and the PFS was 11.8 months. All these studies indicate the potential synergetic effect of MWA and PD-1 antibody.

New perspectives could also be focused on local drug injection, that could also be combined to locoregional treatments, highlighting apoptotic effects, as reported in early-stage NSCLC with the combination of RFA and intratumoral chemotherapy (14, 15). Huang et al. explored the combination of MWA and intratumoral chemotherapy and a median PFS of 8.1 months was achieved.

## Pulmonary metastases

Pulmonary metastases from colorectal cancer (CRC) were commonly observed in clinical practice. Local treatments such as MWA was proved to be an effective treatment in oligometastases disease (16, 17). Han et al. conducted a long-term follow up of patients with CRC pulmonary metastases underwent MWA, the median OS was 76 months and the 5-year survival rate reached as high as 51.6%. The application of MWA provided long-term survival benefit for CRC pulmonary metastases.

## Technical aspects

MWA is mainly performed under CT-guidance. In the last years, other image-guidance modalities were also explored. Cone-beam computed tomography (CBCT) has already been referred to several applications in interventional oncology such as renal cancer and osteoid osteoma (18, 19). It allows 3D images on a selected volume to be produced usually with a relatively small field of view (FOV). Wang et al. explored CBCT-assisted secondary pulmonary tumors ablation and verified that CBCT guided thermal ablation and helical tomotherapy provided comparable clinical effects and safety.

MR-guided MWA for lung malignant tumor could also be another option, just previously explored in rabbits (20, 21). Lin et al. firstly explored the efficacy and safety of MR-guided MWA for lung cancer and showed that the treatment was feasible and efficient. It could be a new guided method for lung cancer patients, even if its applications is limited by the longer procedural time and needed of dedicated devices. From a technical point of view, artificial pneumothorax and artificial hydrothorax were the common applied techniques to treat patients with tumors localized in the subpleural zone (22, 23). To show the effective puncture path and to obtain a sufficient safety margin, combination of artificial pneumothorax and artificial hydrothorax could improve the safety and the complete ablation rate, reducing procedural complications also in the treatment of lung cancer adjacent to vital organs.

## Intraprocedural management

Besides assistance of artificial pneumothorax and artificial hydrothorax, another treatment regimen for patients with tumors localized in the subpleural those patients could be a local pleural anesthesia (24), that can also reduce the patient’s pain and complications.

Thoracic Paravertebral Block (TPB) is generally used in thoracoscopic surgery as a major anesthesia method (25). When a single ultrasound-guided TPB with a large volume of anesthetic is applied during MWA procedure, an effective analgesia can be achieved. This technique allows to increase patient collaboration in order to reduce multiple lung punctures and consequent associated injury.

## Image evaluation

A topic that could be interesting in the treatment of lung tumors with MWA is also represented by the ability to predict and evaluate the treatment efficacy. In detail, apparent diffusion coefficient (ADC) of MRI calculated 24 hours after MWA of lung cancer could be effectively used to predict the early treatment efficacy (20).

Furthermore, Chen et al. showed MRI manifestations of thermal ablation in VX2 tumor of rabbit lung have certain characteristics with strong pathological association. CT combined with MRI multimodal radiomics could also provide an effective new method for clinical evaluation of the immediate efficacy of thermal ablation of lung tumors.

## Conclusions

Locoregional ablative therapies are minimally invasive procedures with an emerging and increasing role for the management of primary and secondary lung tumours.

Factors that could contribute to increase safety and efficacy include a thorough knowledge of new devices and techniques, as well as imaging-guiding modalities, assistive techniques for percutaneous treatment, and the role of prediction and follow-up. Furthermore, combination of percutaneous treatment option, as MWA, with other treatments could also allow a significant improvement of oncological outcomes in patients with both primary and secondary lung cancers.

## Author contributions

All authors listed have made a substantial, direct, and intellectual contribution to the work and approved it for publication.
